# Clinical validation of controlled exposure to house dust mite in the environmental exposure unit (EEU)

**DOI:** 10.1186/s13223-021-00536-3

**Published:** 2021-03-26

**Authors:** Lubnaa Hossenbaccus, Sophia Linton, Jenny Thiele, Lisa Steacy, Terry Walker, Crystal Malone, Anne K. Ellis

**Affiliations:** 1 Department of Biomedical and Molecular Sciences, Queen’s University, Kingston, ON Canada; 2 Allergy Research Unit, Kingston Health Sciences Center - KGH Site, Kingston, Canada; 3 Department of Medicine, Queen’s University, Kingston, ON Canada

**Keywords:** Allergic rhinitis, Environmental exposure unit, House dust mite, Controlled allergen challenge facility

## Abstract

**Rationale:**

The Environmental Exposure Unit (EEU), a controlled allergen exposure model of allergic rhinitis (AR), has traditionally utilized seasonal allergens. We sought to clinically validate the use of house dust mite (HDM), a perennial allergen, in the HDM-EEU, a specially designed facility within the larger EEU.

**Methods:**

Forty-four HDM-allergic and eleven non-allergic participants were screened and deemed eligible for one of two 3-h exposure sessions in the HDM-EEU. Participants were exposed to a modest or higher HDM target, with blood and nasal brushing samples collected before and after allergen exposure. Symptomatic data, including Total Nasal Symptom Score (TNSS), Total Ocular Symptom Score (TOSS), Total Rhinoconjunctivitis Symptom Score (TRSS), and Peak Nasal Inspiratory Flow (PNIF) were collected at baseline, every 30 min until 3 h, on an hourly basis for up to 12 h, and at 24 h following the onset of HDM exposure.

**Results:**

The modest and higher HDM target sessions respectively featured cumulative total particle counts of 156,784 and 266,694 particles (2.5–25 µm), Der f 1 concentrations of 2.67 ng/m^3^ and 3.80 ng/m^3^, and Der p 1 concentrations of 2.07 ng/m^3^ and 6.66 ng/m^3^. Allergic participants experienced an increase in symptoms, with modest target participants plateauing at 1.5 to 2 h and achieving a mean peak TNSS of 5.74 ± 0.65, mean peak TOSS of 2.47 ± 0.56, and mean peak TRSS of 9.16 ± 1.32. High HDM-target allergics reached a mean peak TNSS of 8.17 ± 0.71, mean peak TOSS of 4.46 ± 0.62, and mean peak TRSS of 14.08 ± 1.30 at 3 h. All allergic participants’ symptoms decreased but remained higher than baseline after exiting the HDM-EEU. Sixteen participants (37.2%) were classified as Early Phase Responders (EPR), eleven (25.6%) as protracted EPR (pEPR), seven (16.3%) as Dual Phase Responders (DPR), and nine (20.9%) as Poor Responders (PR). Allergic participants experienced significant percent PNIF reductions at hours 2 and 3 compared to healthy controls. Non-allergics were asymptomatic during the study period.

**Conclusions:**

The HDM-EEU is an appropriate model to study HDM-induced AR as it can generate clinically relevant AR symptoms amongst HDM-allergic individuals.

## Background

The prevalence of allergic rhinitis (AR) varies between 15 and 50% in different populations and as such, is considered a major public health problem worldwide [1–4]. Furthermore, there is a strong association between AR and asthma whereby up to 85% of asthma patients have AR while 15 to 38% of AR patients have asthma [5, 6]. AR is also closely related to other allergic disease such as atopic dermatitis. 85% of atopic dermatitis patients have rhinitis symptoms [7].

AR can be classified as seasonal (e.g., hay fever) or perennial for which house dust mites (HDMs) are the most common cause for perennial AR [8]. HDMs are microscopic arachnids found in dust and bedding and are thus recognized as indoor allergens. A large proportion of patients with AR and/or allergic asthma, are sensitized to HDM, predominantly *Dermatophagoides farinae* (Der f; American HDM) and *Dermatophagoides pteronyssinus* (Der p; European HDM) [9–11]. The prevalence of sensitization to these mites is reported to be from 8 to 90% in different countries [12].

House dust mite-induced allergic rhinitis (HDM-AR) is an IgE-mediated immune response occurring in the mucosal lining of the nasal cavity, evidenced by a clinical history of rhinitis symptoms (sneezing, nasal pruritis, rhinorrhea, and nasal congestion) and/or ocular symptoms (itchy, teary and red eyes) upon HDM exposure, with a positive skin prick test or nasal provocation test and specific IgE testing [5, 13].

Symptoms of HDM-AR vary from mild to severe depending on the individual and negatively impact social interactions, sleep, and productivity in the workplace [3, 14]. Approximately 93% of moderate-severe AR patients seek treatment from a general physician [15] and 18–60% report uncontrolled symptoms despite treatment [3, 16].

The management of HDM-AR focuses on allergen avoidance and alleviation of symptoms by pharmacotherapy [17]. Allergen immunotherapy (AIT) has been shown to treat HDM-AR with lasting effects after the end of treatment. However, there are no specific guidelines for managing HDM-AR unlike those available for AR in general such as Allergic Rhinitis and its Impacts on Asthma (ARIA) [5]. Of note, many of the standard pharmacological agents for AR have not been tested specifically in the context of HDM allergy and many HDM-allergic patients achieve only poor to moderate symptom control [17, 18]. This evidence gap may be relevant as to less-than-adequate control or frequent recurrence of symptoms, given the potential for varying responses to different medications [17, 19].

To study AR pathophysiology, mechanisms, and treatment strategies various research models can be applied. Controlled allergen challenge facilities (CACF) are one such example and are precise, replicable models that provide valuable insights into the mechanisms and kinetics of AR therapeutics, with direct clinical relevance. Other CACFs, including the Vienna Challenge Chamber (VCC), the Fraunhofer allergen challenge chamber, the Strasbourg Experimental Exposure Chamber (EEC) and Biogenics Research Chamber have previously evaluated the use of HDM [20–23]. The Environmental Exposure Unit (EEU) was the first CACF to be built in North America and is currently located in the Kingston Health Sciences Centre–KGH site. It is a validated, internationally recognized model used to study allergic rhinitis (AR) pathophysiology and treatment efficacy. Recently, a standalone house dust mite room, termed the HDM-EEU, was designed and erected within the main EEU for use with perennial allergens. This 760 sq. ft room can host between 5 and 45 participants per session. Particles are delivered into the HDM-EEU in a controlled manner using a particle feeder system with fans guiding the particulate-laden air within the space to allow for an even distribution. Preliminary findings in the HDM-EEU without participants have confirmed that the dispersal equipment and fan setup orientation can effectively distribute HDM particles and that the particle sampling methods capture particle concentrations within the facility [24].

In order to explore and determine the clinical validity of HDM delivery in the HDM-EEU, modest and higher target concentrations of HDM were compared to evaluate the potential impact these differences may have on symptom scoring. This study aimed to clinically validate the use of HDM within the HDM-EEU and additionally to determine the optimal amount of HDM required to produce clinically relevant allergic symptoms in HDM-allergic participants, mimicking “real-life” experiences of AR.

In this study, we report the results of our clinical evaluation of the use of Der p 1 and Der f 1 HDM allergens in inducing nasal and respiratory symptoms in HDM-allergic participants using the HDM-EEU.

## Methods

The protocol for this study was reviewed and ethical clearance was provided by the Queen’s University Health Sciences and Affiliated Teaching Hospitals Research Ethics Board. All study participants provided written, informed consent prior to any study related procedures.

### Participants

Participants on file from previous enrollment with Kingston Allergy Research studies were approached to join the study. Inclusion criteria comprised of the participants to be males or females between the ages of 12 and 65 years old, with a minimum 2-year history of rhinoconjunctivitis symptoms to HDM, and a positive skin prick test (SPT) to house dust mite allergens (*D. pteronyssinus, D. farinae*) confirmed by a wheal diameter of at least 5 mm larger than that produced by the negative control. The participants had to be able and willing to provide written informed consent and be willing to comply with the study requirements. Adolescent participants provided assent, with their parent/guardian reviewing the informed consent form. Women of childbearing potential were required to be abstinent or use a medically acceptable method of birth control throughout the study and produce negative urine pregnancy tests at screening and prior to entering the HDM-EEU. Participants who were experiencing upper respiratory tract infections within 7 days of the HDM exposure visit, had HDM-induced asthma, were unable to adhere to the specified washout periods for medications (Table 1), or who were currently on allergen-specific immunotherapy were excluded from this study. Participants were also excluded if they had a history of drug or alcohol abuse or were known to have positive test results for Hepatitis B, Hepatitis C, HIV, or tuberculosis (unrelated to vaccination). Healthy, non-allergic volunteers were subjected to the same criteria, except that a negative SPT result was required for the entire panel of allergens.

**Table 1:** Medication washout periods

Medication	Washout period
Beta-blockers, alpha-adrenoceptor blockers	Not permitted
Topical alpha-adrenergic agonists	48 h
Short-Acting Antihistamines (e.g. Diphenhydramine)	*3 days
Anti-allergic eye drops (ocular antihistamines, decongestants and cromoglycates)	5 days
Long-Acting Antihistamines (e.g. fexofenadine, loratadine, cetirizine)–note that H2 antagonists (e.g. Zantac, ranitidine) are not considered antihistamines for the purposes of this study	*5 days
Anticholinergics	7 days
Topical Corticosteroids (except 1% or less of hydrocortisone)	7 days
Intranasal or inhaled corticosteroids (e.g. triamcinolone acetonide, fluticasone)	**14 days
Intranasal or inhaled cromolyn	14 days
Leukotriene inhibitors	14 days
Tricyclic Antidepressants and Monoamine oxidase inhibitors	14 days
Systemic corticosteroids (oral)	30 days
Depot corticosteroids	60 days
Biologics	6 months

^*^Prior to skin prick testing and screening nasal brushing

^**^Prior to screening nasal brushing

Study design.

**Fig. 1:**
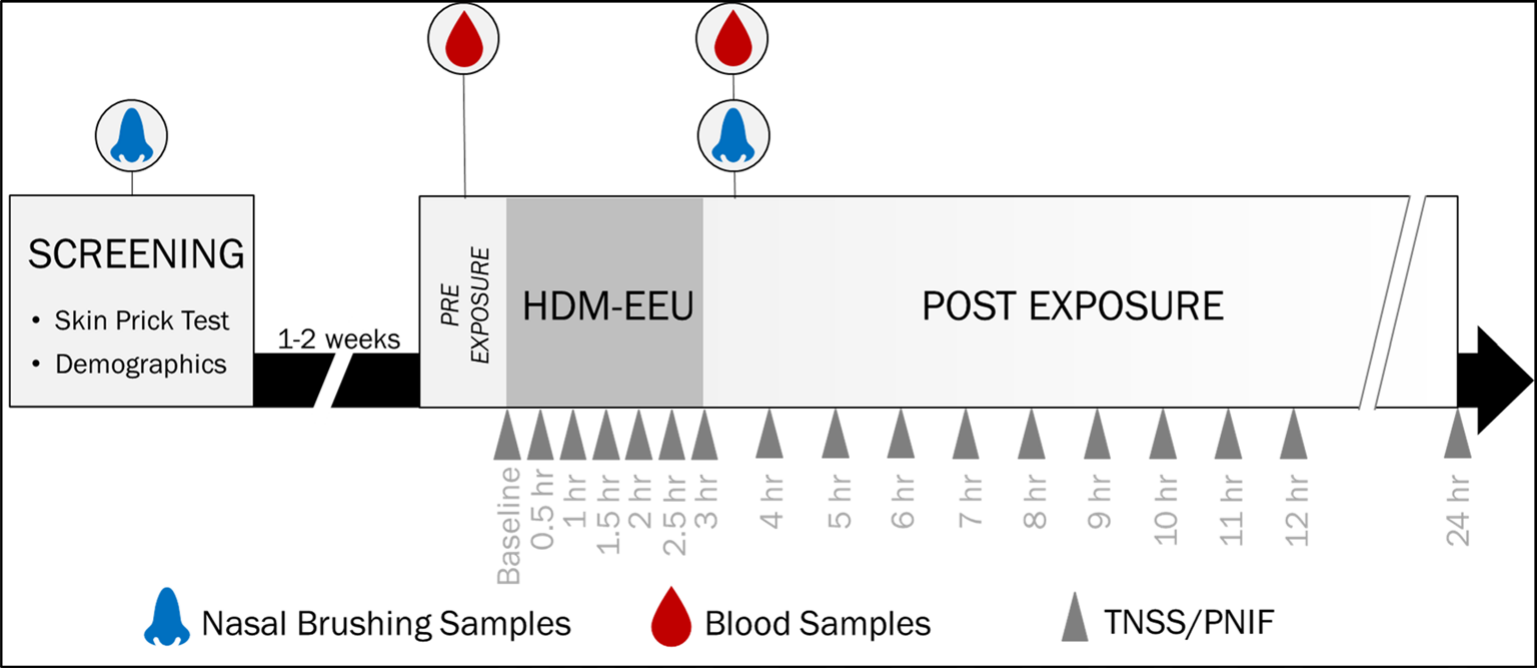
Study design and sample collection. Participants attended an initial screening visit where consent was acquired and SPTs were completed. Eligible participants returned for a 3-h HDM-EEU visit. Pre- and post-exposure blood and nasal brushing samples were collected, but not reported here

As illustrated in Fig. 1, participants initially attended a screening visit where written informed consent was acquired. The following procedures were also completed: review of medical history, measurement of height, weight, and vital signs, allergen panel skin testing (*D. pteronyssinus*, *D. farinae*, timothy grass, ragweed, birch, dog, cat, oak, alder, *Alternaria*), physical and nasal examinations, and urine pregnancy testing (for women of childbearing potential only). Baseline nasal brushing samples were collected at this time.

Eligible participants were invited back 1–2 weeks after screening. They were randomly divided into two groups and attended one of two 3-h HDM-exposure sessions in the HDM-EEU, either one with a modest allergen concentration target or one with a higher allergen concentration. Participants were asked to record their symptoms on paper diary cards, first prior to the onset of allergen exposure (baseline), and subsequently on a half-hour basis throughout the session. Individual nasal and ocular symptoms, including rhinorrhea, sneezing, nasal congestion, nasal itching, itchy/watery eyes, red/burning eyes, and itching of the ears/palate/throat were ranked from 0 to 3, increasing in severity (Table 2). Nasal symptoms (runny nose/nasal drip, nasal congestion/stuffiness, sneezing, and itchy nose) were tallied as Total Nasal Symptom Score (TNSS; max. 12), ocular symptoms (itchy/gritty eyes, watery/tearing eyes, and red/burning eyes) as Total Ocular Symptom Score (TOSS; max. 9), and both nasal and ocular symptoms as well as ear/palate/throat itching as Total Rhinoconjunctivitis Symptom Score (TRSS; max. 24). Participants were also trained to record their Peak Nasal Inspiratory Flow (PNIF), at baseline and every 30 min while in the HDM-EEU, using the In-Check meter (Clement Clark International Ltd, Essex, UK). Participants were trained prior to entering the HDM-EEU and physicians were present to ensure accurate techniques were being employed.

**Table 2:** Ranking of nasal and ocular symptoms

Symptom	Score			
	0	1	2	3
TRSS	Symptom is completely absent	Symptom is present, but not bothersome	Symptom is bothersome, but tolerable	Symptom is hard to tolerate, desiring treatment
TNSS				
Runny nose/ nasal drip				
Nasal congestion/ stuffiness				
Sneezing				
Itchy nose				
TOSS				
Itchy/ gritty eyes				
Watery/ tearing eyes				
Red/ burning eyes				
Ear/ palate/ throat itching				

Participants rated their symptoms on a scale from 0 to 3, increasing in severity on paper diary cards. The individual symptom scores for each participant were tallied as Total Nasal Symptom Score (max 12), Total Ocular Symptom Score (max 9) and a Total Rhinoconjunctivitis Symptom Score (max 24)

Participants were seated in the HDM-EEU for the duration of the exposure period. Upon completion of the study visit, they were provided with a package of take-home diary cards to continue recording symptoms and PNIF on an hourly basis until 12 h post-onset of allergen, as well as at 24 h. The completed diary cards were to be mailed back to the study site.

Further biological samples, including nasal brushing samples of epithelial cells and peripheral blood for PAX gene analysis, CBC differentials, and serum analyses were collected before and after HDM exposure. These results are not reported here.

EEU methodology.

**Fig. 2:**
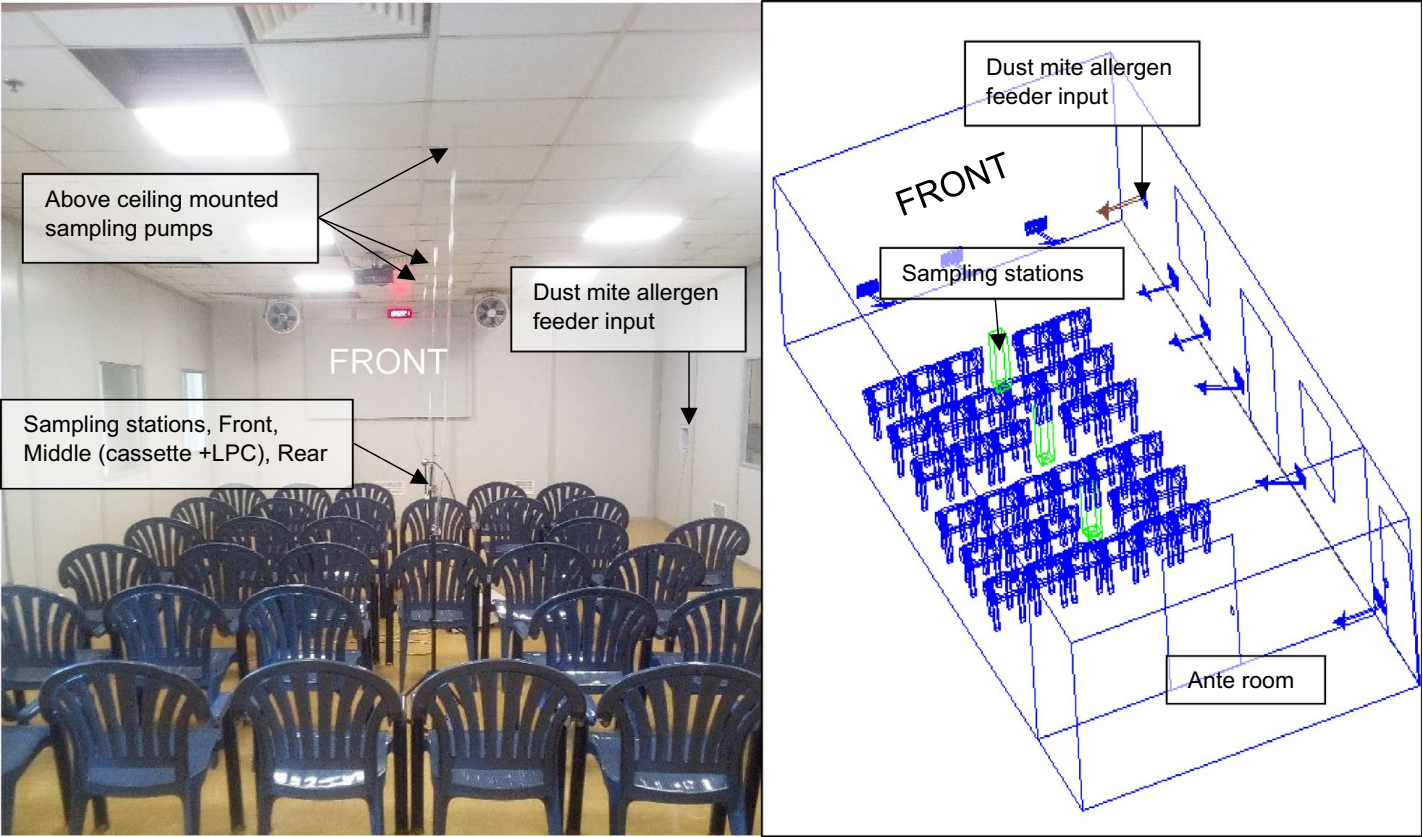
Layout of the House Dust Mite-Environmental Exposure Unit (HDM-EEU). The HDM-EEU is a specially designed unit located within the EEU. It can host up to 45 participants at one time and allows for control of variables, including but not limited to, temperature, humidity, and allergen type and concentration

As described above, the HDM-EEU is a specially designed facility within the EEU at the Kingston Health Sciences Centre–KGH site. The HDM-EEU setup, including location of chairs, feeder, fans, and three 37 mm air sampling stations, is illustrated in Fig. 2. Particle counts at the middle sampler location were measured in real-time using a laser particle counter (LPC) to measure particle count consistency over time. The LPC recorded particle sizes of 2.5 µm, 5.0 µm, 10.0 µm, 15.0 µm, 20.0 µm, and 25.0 µm for the duration of the 3-h HDM exposures. Three above-ceiling mounted GilAir5® sampling pumps running at 4.5 L/min sampled through three 37 mm, Zefon® sampling cassettes, located at the front, middle, and back within the HDM-EEU, were used to sample overall particle levels in the room. The 37 mm sampling cassette filters were transferred into 5 ml Polystyrene tubes with sterile forceps and 2 ml of extraction buffer (Dulbecco’s Phosphate-Buffered Saline (Life Technologies) and 0.05% Tween-20 (MP Biomedicals LLC)) was added to the tubes. Der p1/Der f 1 and total protein concentrations were determined via ELISA (Indoor Biotechnologies Inc.) and Pierce™ BCA Protein Assay (Thermo Fisher Scientific) respectively. HDM allergen was sourced from ALK-Abelló (Post Falls, ID) and contained 50 ng endotoxin per ug Der p or Der f.

### Statistical analysis

The statistical software GraphPad Prism 8.31 (San Diego, CA, USA) was used for data analyses. Results for TNSS, TOSS, TRSS, and PNIF from HDM-allergic and non-allergic participants were evaluated using two-way repeated measures ANOVA with Bonferroni’s correction. Given that natural variation exists in raw PNIF scores, the percentage change in PNIF from baseline was also used to investigate nasal patency. Participants with missing or incomplete diary cards were excluded from the analysis.

## Results

A total of sixty-eight participants were screened for enrollment, with forty-four HDM-allergics and eleven non-allergics deemed eligible for study participation. Twenty allergics and five non-allergics attended the visit with a modest HDM concentration while twenty-four allergics and six healthy controls were in the higher HDM target exposure. One allergic and one non-allergic participant were excluded from the statistical analysis due to missing or incomplete diary cards.

Total Nasal and Symptom Score (TNSS), Total Ocular Symptom Score (TOSS), and Total Rhinoconjunctivitis Symptom Score (TRSS).

**Fig. 3:**
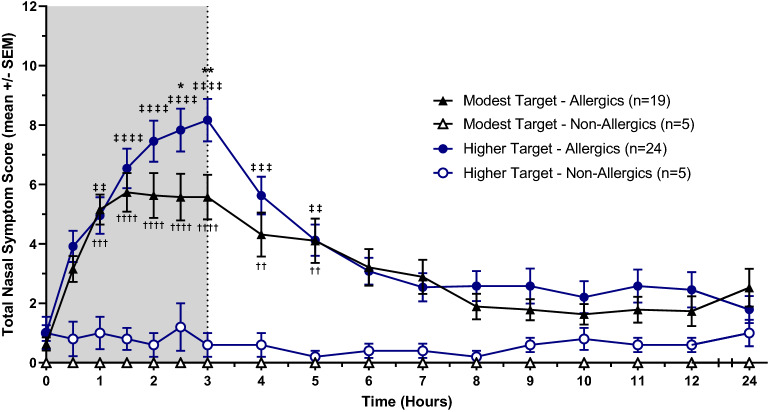
Mean Total Nasal Symptom Score (TNSS). HDM-allergic participants experienced an increase of nasal symptoms beginning from baseline (t = 0). Allergics exposed to a modest HDM target experienced a plateau in symptoms around 1.5 h following the onset of allergen exposure. Allergic participants exposed to a higher HDM target experienced a significantly greater peak in mean TNSS at 2.5 (p < 0.05) and 3 h (p < 0.01) compared to modest target allergics. Comparisons between modest target allergics and non-allergics are represented by “†”, high target allergics and non-allergics by “‡” and modest and high target allergics by “*”. †/‡/* = p < 0.05, ††/‡‡/** = p < 0.01, †††/‡‡‡/*** = p < 0.001, ††††/‡‡‡‡/**** = p < 0.0001

**Fig. 4:**
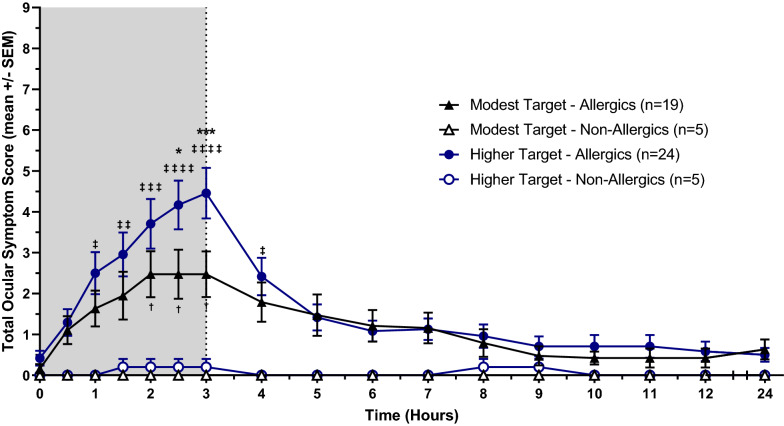
Mean Total Ocular Symptom Score (TOSS). Ocular symptoms (max 9) were significantly greater for HDM-allergics exposed to a higher HDM target from hours 1 to 4 compared to healthy controls. Comparisons between modest target allergics and non-allergics are represented by “†”, high target allergics and non-allergics by “‡” and modest and high target allergics by “*”. †/‡/* = p < 0.05, ††/‡‡/** = p < 0.01, †††/‡‡‡/*** = p < 0.001, ††††/‡‡‡‡/**** = p < 0.0001

**Fig. 5:**
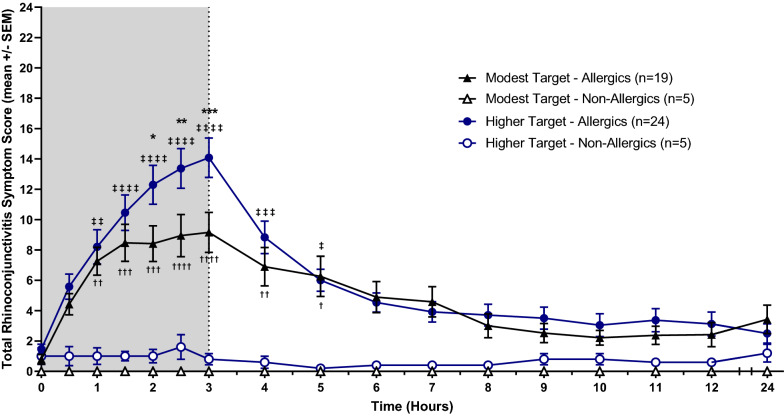
Mean Total Rhinoconjunctivitis Symptom Score (TRSS). All of the participants’ ranked symptoms were tallied as the TRSS (max 24). A steady increase in symptoms were observed for all allergic participants, peaking at 3 h, though those exposed to a higher HDM target achieved a mean peak TRSS of 14.08 ± 1.30 compared to 9.16 ± 1.32 for those exposed to a moderate HDM target. Comparisons between modest target allergics and non-allergics are represented by “†”, high target allergics and non-allergics by “‡” and modest and high target allergics by “*”. †/‡/* = p < 0.05, ††/‡‡/** = p < 0.01, †††/‡‡‡/*** = p < 0.001, ††††/‡‡‡‡/**** = p < 0.0001

Baseline TNSS, TOSS, and TRSS values were comparable, with no significant differences between allergic and non-allergic participants exposed to both modest and higher HDM targets. Allergic participants exposed to a modest HDM target experienced a steady increase in symptoms following the onset of allergen exposure until 1.5 to 2 h, when their nasal, ocular, and total symptoms plateaued. Peak symptomatic values of the participants’ mean reported scores (mean ± standard error) were as follows: TNSS = 5.74 ± 0.65 (Fig. 3), TOSS = 2.47 ± 0.56 (Fig. 4), and TRSS = 9.16 ± 1.32 (Fig. 5). In comparison, HDM-allergics exposed to a higher allergen target experienced a steep increase in symptoms for the entire duration of HDM exposure, peaking at 8.17 ± 0.71 (TNSS), 4.46 ± 0.62 (TOSS), and 14.08 ± 1.30 (TRSS) at 3 h. After exiting the HDM-EEU, all allergic participants experienced a gradual decrease in symptoms, though did not return to baseline by 24 h, at which point TNSS, TOSS, and TRSS were respectively 2.53 ± 0.64, 0.63 ± 0.24, and 3.42 ± 0.94, for modest target participants and 1.79 ± 0.45, 0.50 ± 0.17, 2.40 ± 0.64 for higher target participants.

Allergics in the high target group had significantly elevated TNSS at 2.5 (p < 0.05) and 3 h (p < 0.01), TOSS at 2.5 h (p < 0.01) and 3 h (p < 0.001), and TRSS at 2 h (p < 0.05), 2.5 h (p < 0.01), and 3 h (p < 0.001) compared to modest target allergics. Compared to healthy controls, allergics experienced significantly elevated TNSS and TRSS from 1 to 5 h following the onset of allergen exposure, irrespective of allergen concentration. In comparison, HDM-allergic participants exposed to a higher target of HDM experienced significantly sustained ocular symptoms from 1 to 4 h, whereas those exposed to a modest target experienced significantly elevated TOSS from hours 2 to 3 compared to non-allergic participants (Fig. 4).

### AR Phenotypes

**Fig. 6:**
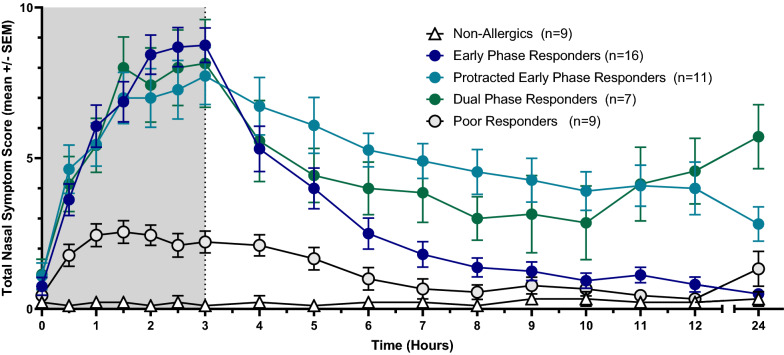
AR phenotypes. HDM-allergic participants were categorized based on their TNSS profiles, as previously described. Sixteen participants (37.2%) were Early Phase Responders, eleven (25.6%) were protracted EPR (pEPR), seven (16.3%) were classified as Dual Phase Responders (DPR) and nine (20.9%) were classified as Poor Responders (PR)

HDM-allergic participants were grouped into AR phenotypes based on TNSS profiles using previously defined and published classifications (Fig. 6) [25]. Sixteen participants (37.2%) experienced a gradual rise in TNSS followed by a reduction of 50% from the peak score by the 6th or 7th hour and were classified as Early Phase Responders (EPR). Eleven participants (25.6%) reported a similar gradual increase in symptoms but did not experience a reduction of 50% in symptoms by hours 6 or 7 and were classified as being protracted EPR (pEPR). Seven participants (16.3%) were classified as a Dual Phase Responder (DPR), in that they experienced a 50% reduction in TNSS by the 6th or 7th hour followed by an increase of at least two points thereafter. Nine participants (20.9%) did not reach a TNSS of 4 following exposure to allergen in the EEU and were therefore classified as Poor Responders (PR).

### Peak nasal inspiratory flow (PNIF)

**Fig. 7:**
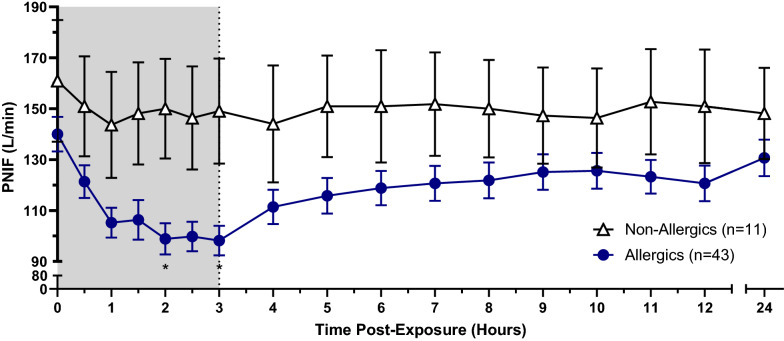
Peak Nasal Inspiratory Flow (PNIF). PNIF values were collected at each timepoint using a PNIF meter. HDM-allergic participants experienced significantly decreased PNIF values at hours 2 (p < 0.05) and 3 (p < 0.01) compared to non-allergic participants. * = p < 0.05

**Fig. 8:**
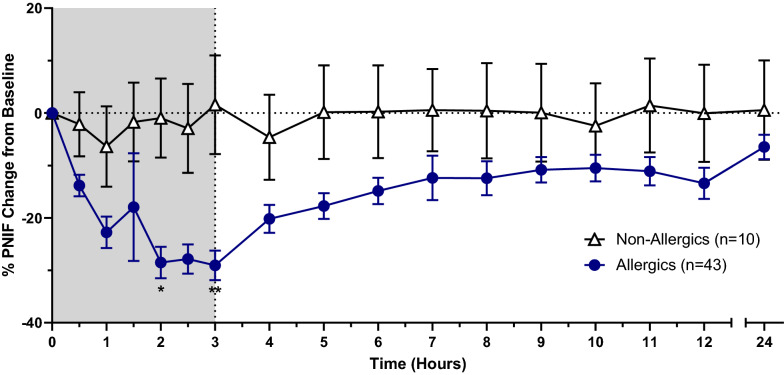
Percent PNIF change from baseline. Precent PNIF change from baseline is a more accurate measure of inspiratory flow as PNIF can be quite variable. HDM-allergic participants had significantly decreased precent PNIF change at hours 2 (p < 0.05) and 3 (p < 0.01) compared to healthy controls. * = p < 0.05, ** = p < 0.01

**Fig. 9:**
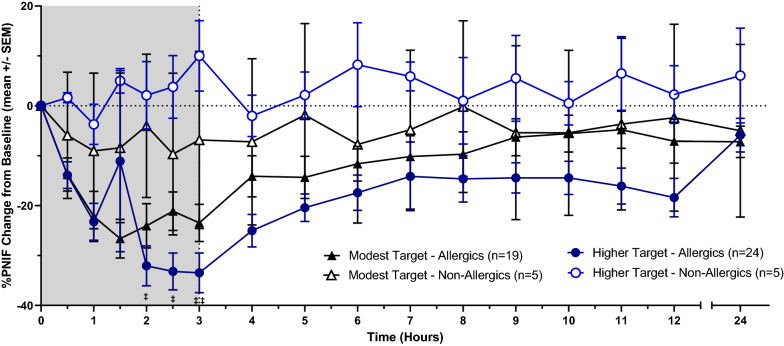
Percent PNIF change from baseline in light of HDM exposure. HDM-allergic participants exposed to a higher HDM target experienced a significant reduction in percent PNIF change from baseline at 2 h (p < 0.05), 2.5 h (p < 0.05) and 3 h (p < 0.01) compared to healthy controls. Allergic participants exposed to a modest HDM target experienced no significant changes in percent PNIF change from baseline relative to control. Comparisons between high target allergics and non-allergics by “‡”. ‡ = p < 0.05, ‡‡ = p < 0.01

**Fig. 10:**
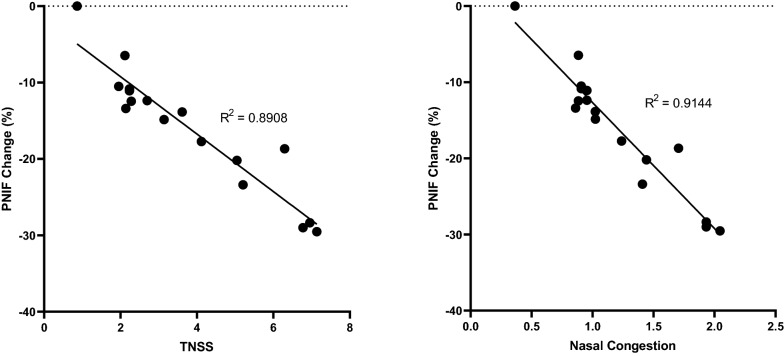
Symptomatic correlations. TNSS, a subjective measure of AR, and percent PNIF change from baseline, a more objective measure, demonstrated a strong correlation (R^2^ = 0.8908) indicating accurate reporting of symptoms from participants. A strong correlation between nasal congestion and percent PNIF change from baseline (R^2^ = 0.9144) also indicate that participants had been properly trained as consistent reporting of symptoms are observed

PNIF recorded by non-allergic participants experienced no statistically significant change compared to baseline. HDM-allergic participants reported a reduction in PNIF given the onset of allergen exposure, though were only significantly different than non-allergics at hours 2 (p < 0.05) and 3 (p < 0.05) (Fig. 7). HDM-allergics similarly experienced significant reductions in percent PNIF change from baseline at 2 (p < 0.05) and 3 h (p < 0.01) following the onset of allergen exposure compared to non-allergic participants (Fig. 8). HDM-allergics exposed to a higher HDM target experienced significant reduction in percent PNIF change from baseline at 2 h (p < 0.05), 2.5 h (p < 0.05) and 3 h (p < 0.01) following the onset of allergen exposure compared to healthy controls, while no significant changes were observed with the modest target allergics (Fig. 9). Strong correlations were observed between TNSS and percent PNIF change from baseline (R^2^ = 0.8908; Fig. 10a) as well as between nasal congestion and percent PNIF change from baseline (R^2^ = 0.9144; Fig. 10b).

### HDM particle data

**Fig. 11:**
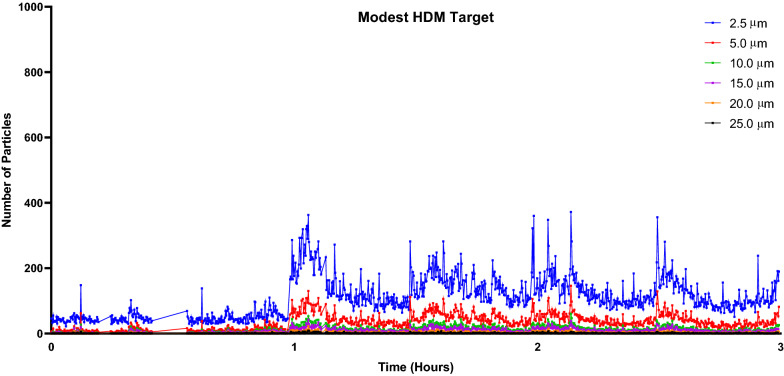
Laser particle counter (LPC) recordings for modest HDM target exposure session. The LPC recorded particles of sizes 2.5 µm, 5.0 µm, 10.0 µm, 15.0 µm, 20.0 µm, and 25.0 µm for the duration of the 3-h exposure. Particles measuring 2.5 µm averaged 108.5 particles

**Fig. 12:**
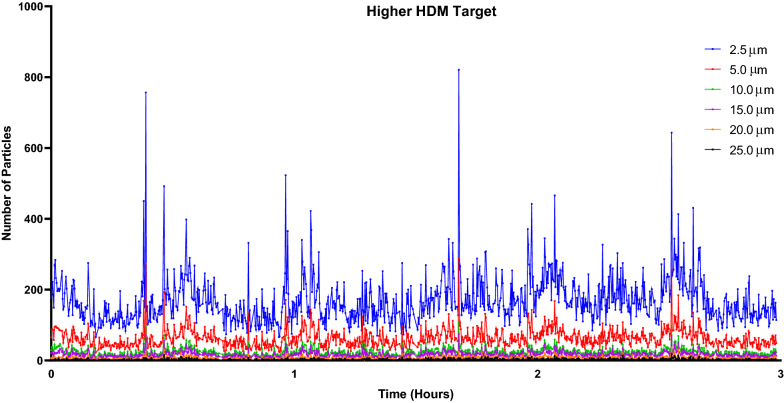
Laser particle counter (LPC) recordings for higher HDM target exposure session**.** The LPC recorded particles of sizes 2.5 µm, 5.0 µm, 10.0 µm, 15.0 µm, 20.0 µm, and 25.0 µm for the duration of the 3-h exposure. Particles measuring 2.5 µm averaged 169.8 particles

**Fig. 13:**
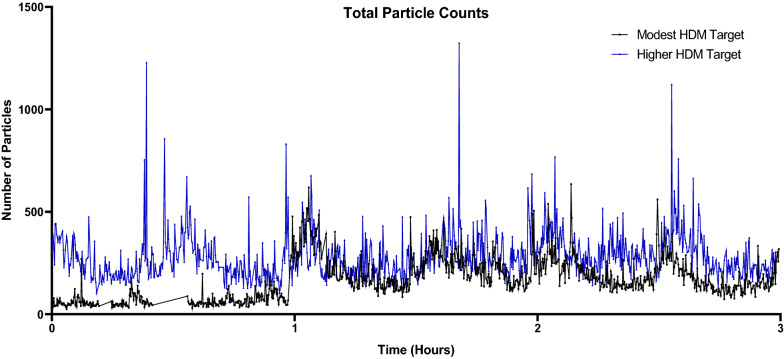
Total Particle Counts**.** Total particle counts (2.5–25 um) over the course of the 3-h HDM exposure sessions demonstrate that the modest HDM target session began at 51 particles and rapidly increased at 1 h to 260 particles. This compares to the higher HDM target which featured a steady flow of allergen exposure, elevated compared to the modest HDM target session

**Fig. 14:**
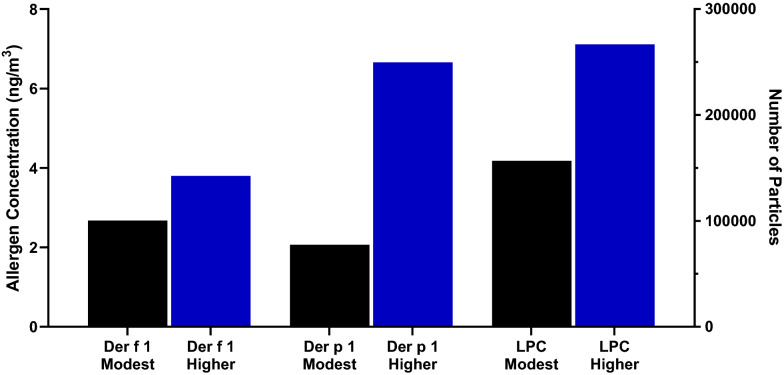
Higher HDM target session had increased Der p 1, Der f 1, and total particle counts than the modest HDM target session**.** The modest HDM target session featured a Der f 1 concentration of 2.67 ng/m^3^, Der p 1 concentration of 2.07 ng/m^3^ while the higher HDM target session involved a Der f 1 concentration of and 3.80 ng/m^3^ and Der p 1 concentration of 6.66 ng/m^3^. The modest HDM target session had a total cumulative particle count of 156,784 while the higher target exposure session featured 266,694 particles

Particles measuring 2.5 µm were most abundant during both HDM-EEU sessions and averaged 169.8 particles per timepoint during the higher HDM target session in comparison to 108.5 particles during the modest session (Figs. 11 and 12). The modest HDM target session began at 51 particles and rapidly increased at 1 h following allergen onset to 260 particles, in comparison to the higher HDM target which featured a steady flow of allergen exposure (Fig. 13). Total particle counts from the LPC mirror findings observed with the ELISA data. The modest and higher HDM exposure sessions respectively featured cumulative total particle counts of 156,784 and 266,694, Der f 1 concentrations of 2.67 ng/m^3^ and 3.80 ng/m^3^, and Der p 1 concentrations of 2.07 ng/m^3^ and 6.66 ng/m^3^ (Fig. 14).

## Discussion

Our results effectively demonstrate that HDM-allergic participants experienced clinically relevant symptoms of AR due to a controlled HDM exposure challenge in the HDM-EEU. The nasal, ocular, and respiratory symptoms experienced by the allergic participants were statistically different than those experienced by healthy, non-allergic participants, the majority of whom experienced no symptoms for the duration of the exposure.

This study featured modest and higher HDM target exposure sessions. Symptomatic responses of allergic participants were generally consistent between the two, with TNSS and TRSS significantly elevated for hours after participants left the HDM-EEU. A single 3-h exposure session also elicited a significant increase in eye-related symptoms of allergic conjunctivitis in allergic participants; however, these were maintained in the higher HDM target allergics to a more significant degree even after exiting the HDM-EEU. Allergic participants exposed to a higher HDM target experienced significantly higher peaks in their mean symptom scores compared to modest HDM target allergics. This indicates that there is a dose-dependent response in allergen exposure and symptom elucidation in the HDM-EEU, similar to the trends observed in the clinical validation of grass pollen in the EEU [26]. As anticipated, the allergic participants’ subjective scoring of nasal congestion correlated well with percent PNIF change from baseline measurements, as well as TNSS, with increased congestion presumably obstructing nasal inspiration, resulting in the lower PNIF values. Such strong correlations suggest that participants were trained effectively to accurately record their symptoms, through both TNSS and PNIF.

Evaluation of allergen concentration in this HDM-EEU study was different than with the seasonal allergens previously used in the EEU primarily due to the fact that the product received from ALK, HDM and their feces, were reduced through grinding and dry sieving. Rotorods^©^ are typically used with pollen to establish live-counts, though with HDM, we optimized an approach with the LPC, which reported a range of particle sizes from 2.5 to 25.0 µm, to make live adjustments and ELISA analyses to confirm the results. Smaller particle sizes are of greater clinical significance as particles less than 10 µm tend to travel farther down the respiratory tract deeper into the lungs [27]. A study investigating the role of HDM particle size in the bronchial response of asthmatics found that large particles (> 10 µm) would elicit early-phase responses without late-phase reactions [28]. As 2.5 µm particles were most abundant in the HDM-EEU, it would be expected that participants would present a more pronounced late phase response, characterized by a DPR. On the contrary, though similar to previous EEU clinical validations studies, most participants exposed to HDM were EPRs or pEPRs, while the DPR phenotype has consistently been observed to be the least prevalent. Our findings would suggest that the allergic participants’ phenotypes are intrinsic. Contrary to other validations, however, we observed a group of HDM-allergic participants who were phenotyped as “Poor Responders”. Despite demonstrating clinical history for HDM-AR and positive skin prick test results to Der p and Der f allergen extracts, the TNSS of roughly 20% of our allergic participants did not reach or surpass 4. Whether this is a dose-related response or an innate biological adaptation to persistent allergen exposure is unclear and necessitates further investigation.

As aforementioned, there are several CACFs that have performed a clinical validation using HDM. These chambers vary in terms of their HDM material, time of exposure, allergen delivery, and allergen concentrations, making comparisons between CACF’s difficult. In general, allergen concentrations of other HDM validation studies range between 15 and100 ng/m^3^ [20–23]. The air during the HDM-EEU exposure sessions was sampled using filters and the Der p 1 and Der f 1 concentrations were evaluated using an ELISA. The levels of Der f 1 and Der p 1 were significantly lower in both sessions ranging between 2.67–3.90 ng/m^3^ and 2.07–6.66 ng/m^3^, respectively. Our allergen exposure provides a closer comparison to natural allergen exposure, as airborne concentrations of Der p 1 in living rooms has been reported to be approximately 0.03 ng/m^3^. However, certain activities, such as making the bed, was associated with increased allergen concentrations, roughly 30 ng/m^3^ [29]. In our study, it is important to take into consideration the effectiveness of achieving a TNSS of 6, which is often the standard for CACF studies [30]. We found that the higher HDM target featuring a Der p 1 concentration of 6.66 ng/m^3^ and Der f 1 concentration 3.80 ng/m^3^ was more effective at achieving the latter than the modest HDM target. These findings suggest that in the HDM-EEU, lower allergen concentrations are enough to induce clinically relevant and measurable symptoms.

## Conclusions

This clinical validation study confirms the capacity of the HDM-EEU to produce targeted and clinically relevant nasal and respiratory symptoms of AR in HDM-allergic participants compared to healthy controls and confirms that it is an appropriate model to study HDM-AR. An ideal HDM allergen range featuring a Der p 1 concentration ~ 6.66 ng/m^3^ and Der f 1 concentration ~ 3.80 ng/m^3^ was determined to be most effective at elucidating a TNSS of 6. As a result of this validation, use of the HDM-EEU can be extended to investigations of therapies for the treatment of HDM-AR. This will allow for greater understanding of safety, efficacy, and onset and mechanisms of action of HDM-AR medications in a clinically relevant context.

## Data Availability

The data supporting this study are available upon request from the corresponding author.

## Abbreviations

AIT: Allergy immunotherapy
AR: Allergic rhinitis
ARIA: Allergic rhinitis and its impacts on asthma
CACF: Controlled allergen challenge facilities
Der f: *Dermatophagoides farinae*
Der p: *Dermatophagoides pteronyssinus*
DPR: Dual phase responder
EEC: Experimental exposure chamber
EEU: Environmental exposure unit
EPR: Early phase responders
HDM: House dust mite
HDM-AR: House dust mite induced allergic rhinitis
HDM-EEU: House dust mite environmental exposure unit
pEPR: Protracted early phase responders
PNIF: Peak nasal inspiratory flow
PR: Poor responders
TNSS: Total nasal symptom score
TOSS: Total ocular symptom score
TRSS: Total rhinoconjunctivitis symptom score
VCC: Vienna challenge chamber
